# Oligomerization enhances the binding affinity of a silver biomineralization peptide and catalyzes nanostructure formation

**DOI:** 10.1038/s41598-017-01442-8

**Published:** 2017-05-03

**Authors:** Tatsuya Sakaguchi, Jose Isagani B. Janairo, Mathieu Lussier-Price, Junya Wada, James G. Omichinski, Kazuyasu Sakaguchi

**Affiliations:** 1 Laboratory of Biological Chemistry, Department of Chemistry, Faculty of Science, Hokkaido University, Sapporo, 060-0810 Japan; 2 Département de Biochimie et Médicine Moléculaire, Université de Montréal, C.P. 6128 Succursale Centre-Ville, Montréal, QC H3C 3J7 Canada

## Abstract

Binding affinity and specificity are crucial factors that influence nanostructure control by biomineralization peptides. In this paper, we analysed the role that the oligomeric state of a silver biomineralization peptide plays in regulating the morphology of silver nanostructure formation. Oligomerization was achieved by conjugating the silver specific TBP biomineralization peptide to the p53 tetramerization domain peptide (p53Tet). Interestingly, the TBP–p53Tet tetrameric peptide acted as a growth catalyst, controlling silver crystal growth, which resulted in the formation of hexagonal silver nanoplates without consuming the peptide. The TBP–p53Tet peptide caps the surface of the silver crystals, which enhances crystal growth on specific faces and thereby regulates silver nanostructure formation in a catalytic fashion. The present findings not only provide an efficient strategy for controlling silver nanostructure formation by biomineralization peptides, but they also demonstrate that in this case the oligomeric peptides play a unique catalytic role.

## Introduction

Inorganic nanomaterials exhibit significantly different properties relative to their bulk-sized counterparts. In particular, silver nanomaterials are extremely attractive because of their multi-functionality. Silver nanomaterials possess unique optical^1–3^, electrical^4–6^ and catalytic^7, 8^ properties, which are highly dependent on both the size^9^ and shape^9, 10^ of the nanostructure. In addition, we recently reported that the anti-cancer activity of silver nanomaterials is dependent on their nanostructure^11^. Thus, methods that can precisely regulate the size and shape of silver nanostructures are becoming increasingly more important to the development of high-performance materials.

Biomimetic approaches represent an effective mechanism for fabricating nanomaterials because of the self-assembly and specific recognition capacities of biomolecules^12–14^. In particular, biomineralization peptides are often employed in the biomimetic synthesis of nanomaterials due to their intrinsic ability to self-assemble and bind to the surfaces of materials. As a result, a number of biomineralization peptides have been identified and used to prepare a wide variety of functional inorganic nanomaterials^15–17^. Some of these biomineralization peptides have been shown to bind to a specific crystal plane, and thereby control nanocrystal shape by directing a colloidal nanocrystal synthetic process^18^. These specialized biomineralization peptides are typically identified by a systematic optimization of a peptide sequence.

One of the crucial challenges facing peptide–mediated biomineralization is the ability to precisely control both their spatial orientation and their valency. The ability to control these parameters is particularly important considering the impact these parameters have on the resulting nanostructure formation. Peptide-mediated biomineralization is a complex reaction, and numerous factors regulate the process. Several previous studies have been conducted in an attempt to better understand the fundamental properties of this process. In particular, these studies have examined the effect of varying a particular parameter on the structure and morphology of the resultant nanomaterial, including the effects of reductant strength^19^ and pH^20^ on gold biomineralization as well as the effects of buffer composition on palladium biomineralization^21^.

More recently, we demonstrated that controlling the precise topological assignment of a palladium biomineralization peptide results in enhanced catalytic activity of the nanomaterial^22^. In this case, the oligomeric state of the biomineralization peptide was varied using the tetramerization domain of the tumour suppressor protein p53 (p53Tet) as the control element^23, 24^. These results demonstrated that the shape of the nanomaterial reflected the topology of oligomeric biomineralization peptides, and this indicated that the oligomeric biomineralization peptides functioned as scaffolds to control the shape and morphology of the nanomaterial. Thus, these studies clearly demonstrated that it was possible to regulate nanocrystal structure formation by altering the oligomeric state of biomineralization peptides.

Given the importance of silver nanomaterials, it is crucial to understand the key factors governing silver biomineralization. Unfortunately, the number of investigations to date that focus on silver biomineralization have been limited. In this study, we have evaluated the relationship between the peptide-binding affinity for the material surface and the peptide-oligomerization state for silver biomineralization. For these studies, we used a peptide consisting of the silver binding TBP peptide fused to the p53Tet peptide. Our studies demonstrate that the binding affinity and specificity of the TBP–p53Tet silver biomineralization fusion peptide increase through oligomerization, and that the oligomeric biomineralization peptides are able to control nanostructure formation. It was also determined that decreasing either the temperature or the peptide concentration favoured silver nanoplate formation. Interestingly, the TBP–p53Tet silver biomineralization peptide plays a catalytic role during the process as it is not consumed during nanostructure formation. This is in sharp contrast to what we previously observed when using the Pd4–p53Tet palladium biomineralization peptide, which is incorporated into nanostructures at almost 100%. Taken together, the results suggest that the effect of oligomerization on biomineralization peptides can vary tremendously depending on the type of metal ion used during the nanoplate formation process.

## Results

### Peptide Design and synthesis

**Figure 1:**
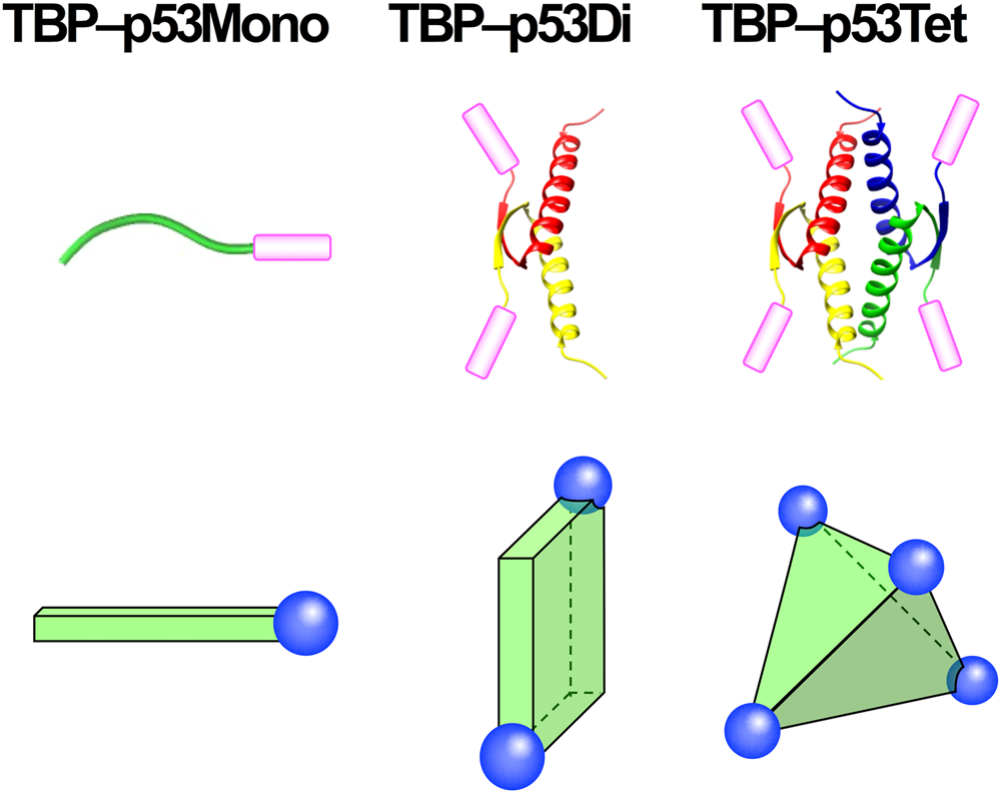
(Top panels) Structures of TBP‒p53 peptides where TBP peptides are illustrated as pink bar. (Bottom panels) The relative positions of the TBP peptides are represented as the blue spheres.

In order to regulate the oligomeric state of a biomineralization peptide, the silver binding TBP peptide^25^ was fused to the tetramerization domain of p53(p53Tet) to produce the TBP–p53Tet peptide. Our working model is that the four p53Tet regions will regulate the relative orientation of the four TBP metal-binding regions so that they are positioned at the vertices of a tetrahedron in the fusion peptide, as shown in Fig. 1. We also prepared dimeric and monomeric forms of the fusion peptide by varying select amino acids within the p53Tet peptide. The dimeric peptide (TBP–p53Di) was prepared by introducing an alanine to threonine substitution at position 347 (A347T) in the p53Tet peptide^26^. The monomeric peptide (TBP–p53Mono) was prepared by changing three hydrophobic amino acids (L330, I332, and L344) in the p53Tet to alanine. These three hydrophobic amino acids contribute several critical interactions required for tetramer formation by p53Tet (Table 1). The oligomerization states of the TBP–p53 peptides were confirmed by both gel filtration chromatography and CD spectroscopy (Figures S1, S2). The stabilities of TBP–p53Tet and TBP–p53Di were determined by generating thermal denaturation curves for each TBP–p53 peptide based on changes in ellipticity at 222 nm in the CD spectra using a two-state transition mode. The denaturation curves for the TBP–p53Tet and TBP–p53Di peptides are shown in Figure S3, and the thermodynamic parameters are summarized in Table S1. The parameters for TBP–p53Tet were almost identical to those for p53Tet, and this indicates that attaching the TBP peptide to p53Tet had no effect on the stability of the p53 tetramer. The oligomer stability of TBP–p53Di was lower than that of TBP–p53Tet, but nearly all of TBP–p53Di formed the oligomer at 20 °C (Table S2).

**Table 1:** Sequences of the TBP–p53 peptides.

Peptides	Sequences
TBP‒p53Mono	H-RKLPDAGGDGEYFTAQARGRERFEMFREANEALELKDAQAGKE-NH_2_
TBP‒p53Di	H-RKLPDAGGDGEYFTLQIRGRERFEMFRELNETLELKDAQAGKE-NH_2_
TBP‒p53Tet	H-GSRKLPDAGGDGEYFTLQIRGRERFEMFRELNEALELKDAQAGKE-OH

### Effects of oligomerization and orientation control of TBP–p53 peptides on the binding specificity to the silver nanostructure

The binding of the TBP–p53 peptides to silver nanoplates and silver nanoparticles were investigated using QCM, and the binding affinity was calculated based on the Langmuir’s adsorption isotherm. In the case of peptide binding to a metal surface, the chemical reaction for binding can be represented as

1 $$P+S\rightleftharpoons PS$$

where PS represents a peptide (P) bound to a surface site on S. The equilibrium constant K for this reaction is given by

2 $$K=\frac{[PS]}{[P][S]}=\frac{{k}_{a}}{{k}_{d}}$$

where k_a_ and k_d_ are the association and dissociation constants, respectively. The coverage of the surface is expressed as *θ*, and the Langmuir’s isotherm dictates that the rate of surface reaction is given by

3 $$\frac{d\theta }{dt}={k}_{a}(1-\theta )c-{k}_{d}\theta $$

where c is the adsorbate concentration. When binding reactions are in equilibrium, equation (3) is 0. In such cases, *θ* can be described as:

4 $$\theta =\frac{Kc}{1+Kc}$$

In, addition, the coverage of the surface (*θ*) is described as *θ* = [PS]/[S]_tot_, where [S]_tot_ is total number of binding sites. From this equation and equation (4),

5 $$\frac{c}{[PS]}=\frac{c}{{[S]}_{tot}}+\frac{1}{K{[S]}_{tot}}$$

**Figure 2:**
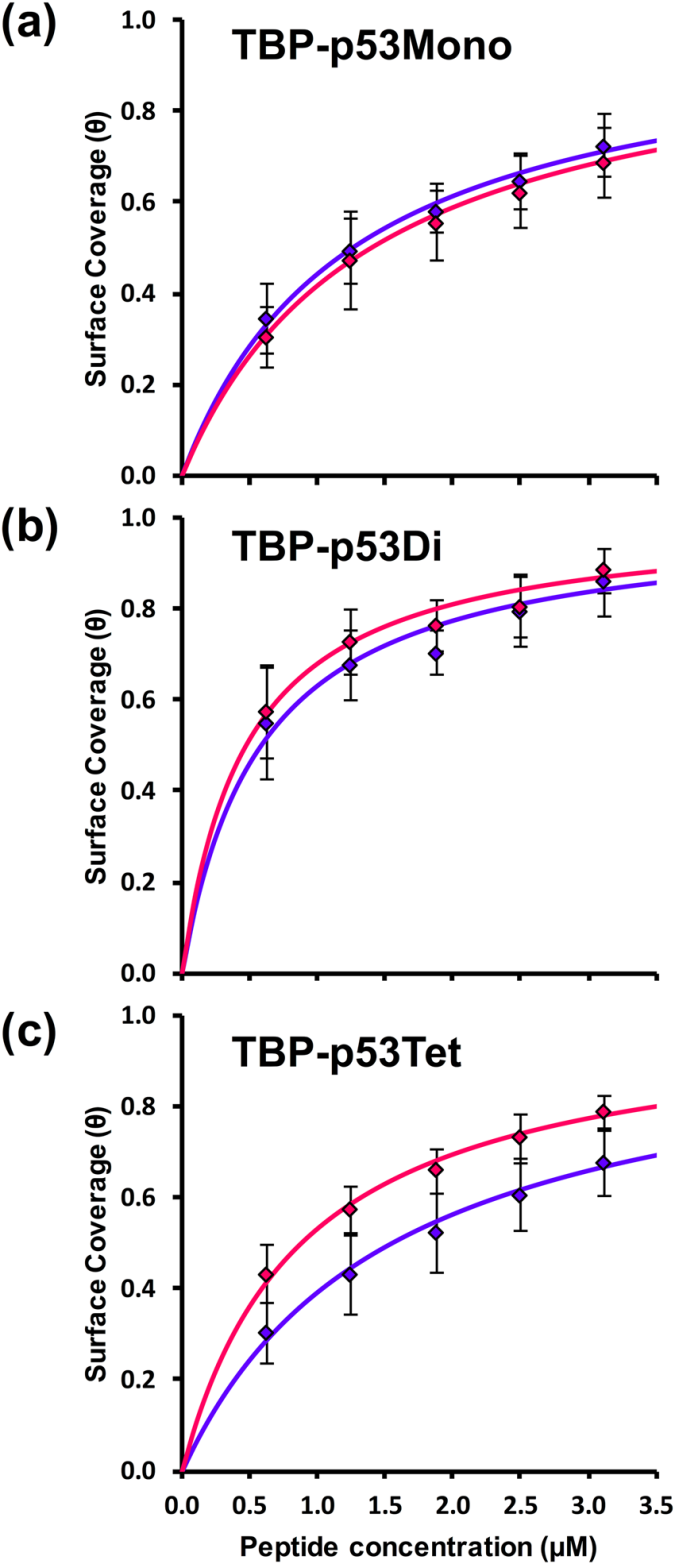
Plot of the surface coverage against peptide concentration of (**a**) TBP‒p53Mono, (**b**) TBP‒p53Di and (**c**) TBP‒p53Tet. The markers indicate observed surface coverage for nanoparticles (blue) and nanostructures (red). The lines indicate calculated surface coverage for nanoparticles (blue) and nanostructures (red). The error bars of each plot are standard deviations.

In this study, *c* corresponds to the concentration of added TBP–p53 peptide, and [*PS*] corresponds to the frequency change of the QCM electrode (*Δf*). Therefore, we calculated the equilibrium constant (*K*) and the total number of binding sites ([*S*]_*tot*_) by plotting *c/*[*PS*] versus peptide concentration (*c*). The calculated equilibrium constant of TBP–p53 peptides absorption to silver nanoparticles and nanoplates are summarized in Table 2. Figure 2 shows the measured surface coverage (*Δf/*[*S*]_*tot*_) and calculated surface coverage using equation (4). The binding affinities of TBP–p53 peptides to silver nanoparticles and silver nanoplates were highly dependent on the oligomerization states of the TBP–p53 peptides. The equilibrium constants of TBP–p53Mono adsorption to silver nanoparticles and silver nanoplates were 0.80 μM^−1^ and 0.72 μM^−1^, respectively. Thus, there is no significant difference between the binding affinities of the monomeric peptide with nanoplates and nanoparticles. In contrast, the oligomeric TBP–p53 peptides (TBP–p53Di and TBP–p53Tet) bound to silver nanoplates more strongly than silver nanoparticles. The equilibrium constants adsorption to silver nanoparticles and silver nanoplates were 1.71 μM^−1^ and 2.11 μM^−1^ for TBP–p53Di, and 0.65 μM^−1^ and 1.74 μM^−1^ for TBP–p53Tet, respectively.

**Table 2:** Equilibrium constants of peptide adsorption for silver nanoplates and nanoparticles.

	K (μM^−1^)		Nanoplates specificity
	Nanoparticles	Nanoplates	
TBP‒p53Mono	0.80 ± 0.21	0.72 ± 0.25	0.90
TBP‒p53Di	1.71 ± 0.59	2.11 ± 0.80	1.23
TBP‒p53Tet	0.65 ± 0.28	1.13 ± 0.21	1.74

Standard errors of mean are indicated.

### Effect of oligomerization state of TBP–p53 peptides on the silver nanostructure formed by biomineralization

**Figure 3:**
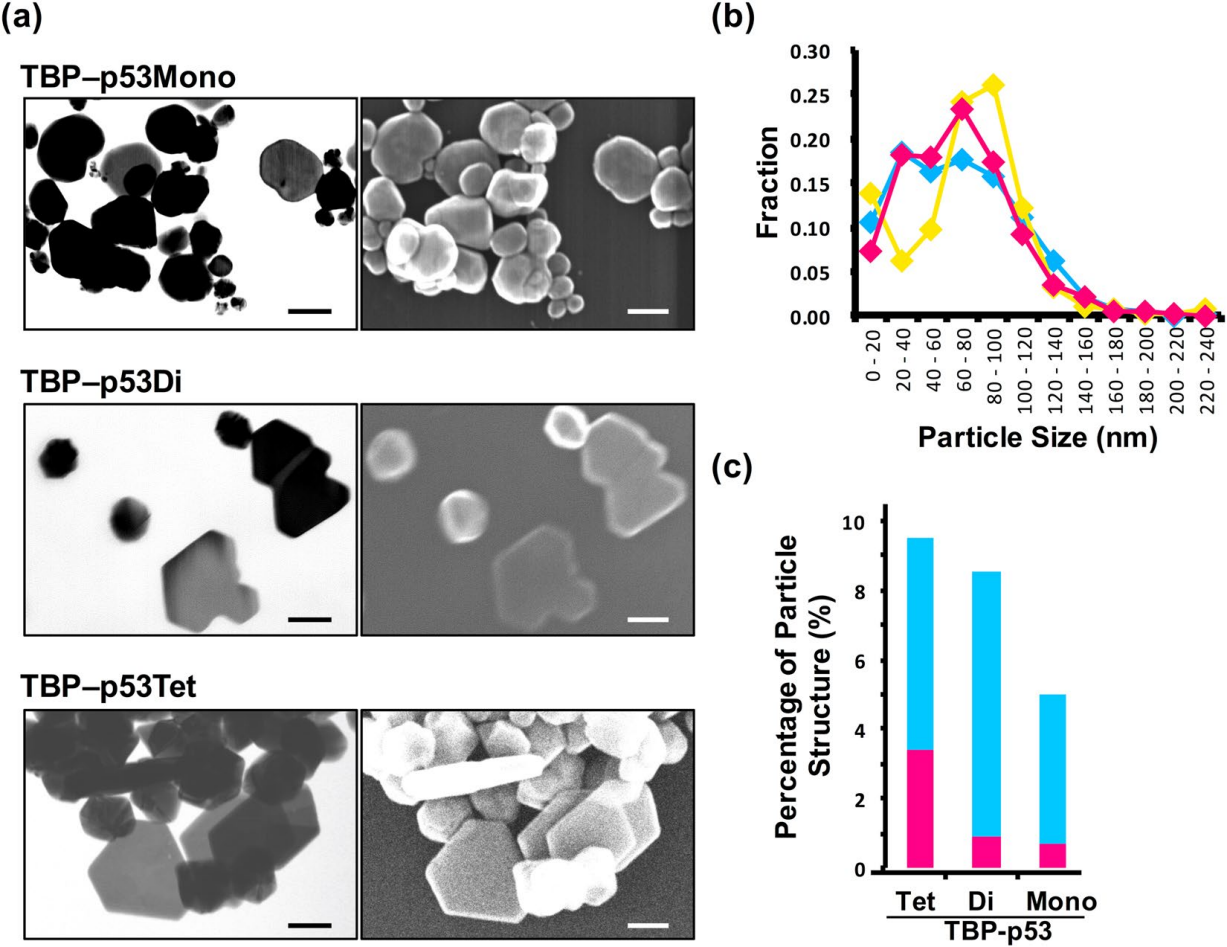
(**a**) Representative electron microscopy images of silver nanostructures formed by using TBP‒p53Mono, TBP‒p53Di and TBP‒p53Tet. Left panels are TEM mode images, and right panels are SEM mode images of STEM. The scale bar is 100 nm. (**b**) Size distribution line graph of silver nanostructures formed by using TBP‒p53Tet (red), TBP‒p53Di (yellow) and TBP‒p53Mono (blue). (**c**) Percentages of silver nanoplates formation by using TBP‒p53Tet, TBP‒p53Di and TBP‒p53Mono. The silver nanoplates were separated into 2 types: symmetric hexagonal plates (magenta) and other shapes(blue).

Silver nanostructures were formed with the TBP–p53 peptides and we promoted the biomineralization reaction using the weak reducing agent L-ascorbic acid at a low temperature in order to gradually grow the nanostructures. The TBP–p53 peptides (10 μM) were incubated with 100 μM silver nitrate for 2 days at 20 °C, and the silver nanostructures were observed by STEM (Fig. 3). The nanostructures formed with TBP–p53Mono were mainly rounded spherical silver nanoparticles, whereas the TBP–p53 peptide oligomers (TBP–p53Di and TBP–p53Tet) formed silver nanoplates. Notably, TBP–p53Di formed crooked polygonal nanoplates, whereas TBP–p53Tet formed highly symmetric hexagonal nanoplates. Disordered and non-uniform nanostructures were formed in the absence of peptides or in the presence of the TBP peptide (Figure S4). The size distribution and the proportion of nanoplates were determined from the STEM images (Fig. 3a). The average size of the nanostructures formed by TBP–p53Mono, TBP–p53Di, and TBP–p53Tet were 67.8 ± 39.1 nm, 77.3 ± 49.7 nm, and 66.6 ± 35.8 nm, respectively, and these values are not significantly different (Fig. 3b). In contrast, the proportion of nanoplates was highly dependent on the oligomeric state of the TBP–p53 peptide, as shown in Fig. 3c. The percentage of hexagonal silver nanoplates was high when the valence of TBP was high, suggesting that TBP–p53Tet is able to bind to a specific surface of silver nanocrystals during the growing process.

### Efficiency of biomineralization reaction

The amount of silver nanostructures formed by the biomineralization reaction were analysed and it was determined that almost all of the silver ions were reduced to Ag^0^ by the biomineralization reaction with each of the TBP–p53 peptides, as shown in Table 3. The proportion of silver nanostructure formed using TBP–p53Mono, TBP–p53Di, and TBP–p53Tet were 98.6 ± 2.0%, 92.2 ± 2.0%, and 98.0 ± 0.2%, respectively. In addition, the consumption of TBP–p53 peptides during the biomineralization reaction was monitored by measuring the peptide concentration in the supernatant of the biomineralization reaction (Table 3). Interestingly, the TBP–p53Tet peptide was not consumed during the biomineralization reaction as essentially 100% remained in the supernatant after the reaction. Similarly, only small amounts of the TBP–p53Mono (21%) and TBP–p53Di (12%) were consumed.

**Table 3:** The formation rate of silver nanostructure and the percentage of remaining peptide after silver nanostructure formation by using TBP‒p53Mono, TBP‒p53Di and TBP‒p53Tet.

Peptides	Ag nanostructure formation (%)	Consumed peptide (%)
TBP–p53Mono	98.6 ± 2.0	21.3 ± 5.8
TBP–p53Di	92.2 ± 2.0	11.6 ± 9.1
TBP–p53Tet	98.0 ± 0.2	−1.4 ± 2.0

Standard errors of mean are indicated.

### Effects of reaction temperature and peptide concentration on biomineralization to form silver nanoplates

**Figure 4:**
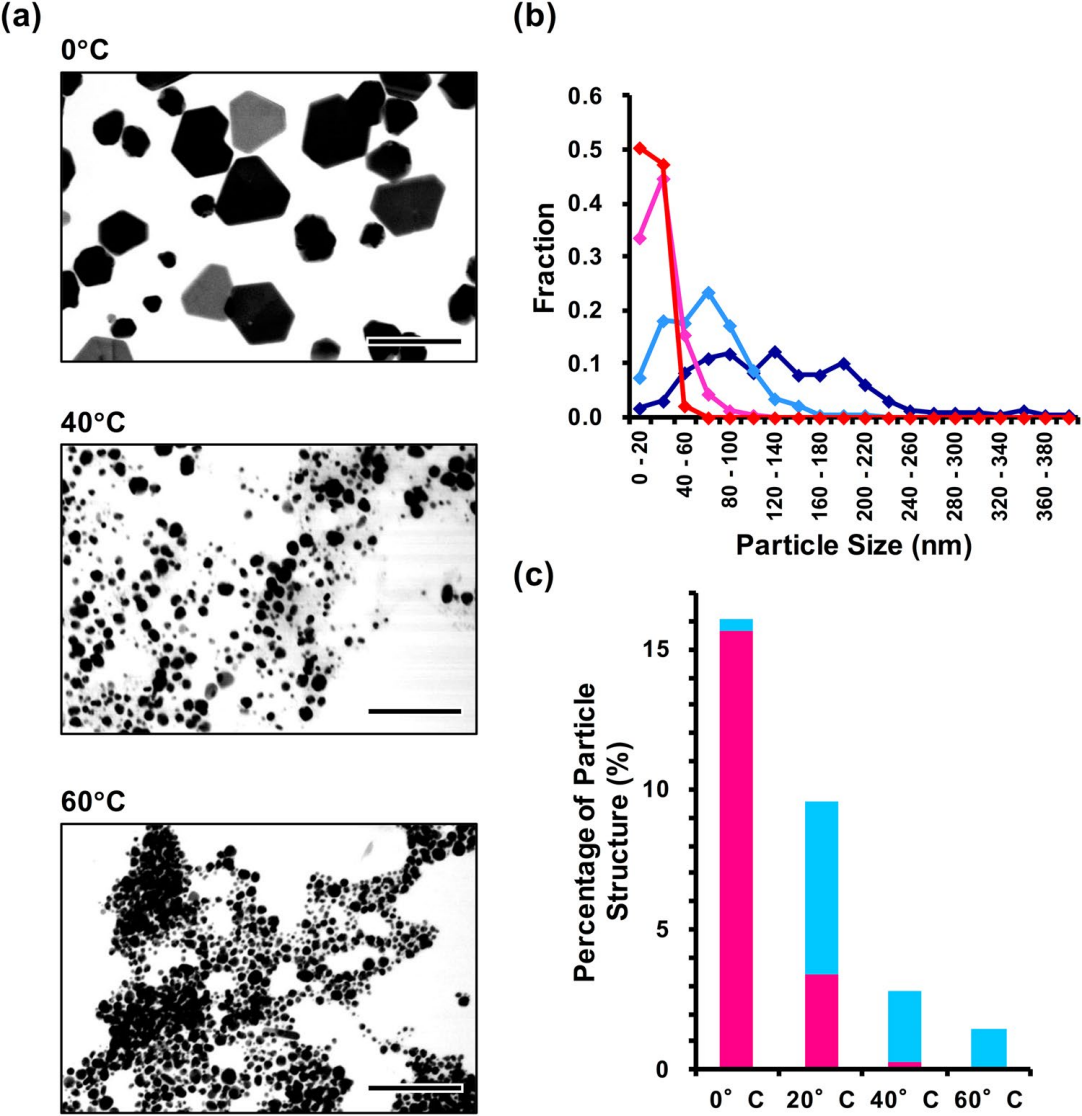
(**a**) Representative TEM images of silver nanostructures formed by using TBP‒p53Tet at 0 °C, 40 °C and 60 °C. The scale bar is 200 nm. (**b**) Size distribution line graph of silver nanostructures formed by using TBP‒p53Tet at 0 °C (purple), 20 °C (cyan), 40 °C (magenta), and 60 °C (red). (**c**) Percentages of silver nanoplates formation by using TBP‒p53Tet at 0 °C, 20 °C, 40 °C, and 60 °C. The silver nanoplates were separated into 2 types: symmetric hexagonal plates (magenta) and other shapes (blue).

The results above demonstrated that the oligomeric TBP–p53 peptides regulate silver nanostructure formation, which leads to the formation of silver nanoplates. Typically, silver nanoplate formation can be improved by using lower reactions temperature because of their thermodynamic instability and low surface energy^27–30^. Therefore, we analysed the effect of temperature on our biomineralization reaction. Silver nanostructure formation by TBP–p53Tet was characterized at 0, 40, and 60 °C. As predicted, the type and size of nanostructures formed were highly dependent on the reaction temperature, as shown in Fig. 4a and b. The proportion of silver nanoplates was higher when the reaction temperature was lower (Fig. 4c), whereas the average size of the nanostructures was smaller and the size distribution was narrower as the reaction temperature was increased.

**Figure 5:**
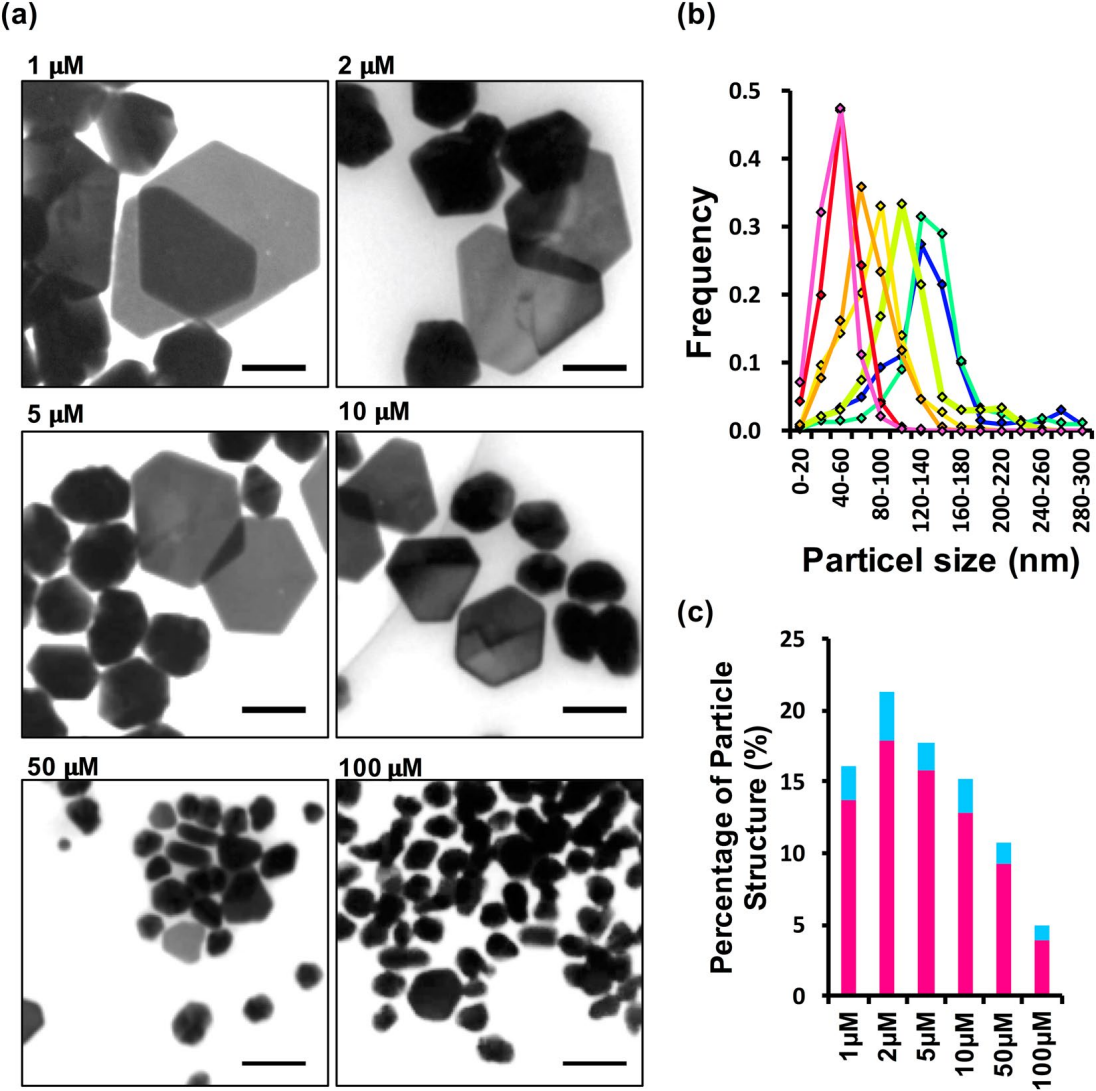
(**a**) Representative TEM images of silver nanostructures formed by using various concentration of TBP‒p53Tet at 0 °C. The scale bar is 100 nm. (**b**) Size distribution line graph of silver nanostructures formed by using 1 μM (blue), 2 μM (blue-green), 5 μM (yellow-green), 10 μM (yellow), 50 μM (red), and 100 μM (magenta) TBP‒p53Tet at 0 °C. (**c**) Percentages of silver nanoplates formation by using various concentration of TBP‒p53Tet at 0 °C. The silver nanoplates were separated into 2 types: symmetric hexagonal plates (magenta) and other shapes (blue).

Next, we analysed the effect of peptide concentration on the silver nanostructures formed during the biomineralization reaction. The biomineralization reactions were performed using concentrations of TBP–p53Tet ranging from 1 to 100 μM at 0 °C. The size and morphology of the silver nanostructures were highly dependent on the peptide concentration (Fig. 5). The proportion of nanoplates was higher at lower peptide concentrations, with the highest proportion at 2 μM TBP–p53Tet (Fig. 5c). In addition, the nanostructure size decreases with increasing peptide concentration (Fig. 5b).

## Discussion

In this study, we have demonstrated that oligomerization of the biomineralization peptide enhances their binding specificity towards the surfaces of nanomaterials and the tetrameric biomineralization peptide acted as a silver nanoplate-growth catalyst. Biomineralization peptide oligomerization leads to silver nanostructure control by regulating the crystal growth process. The oligomerization of biomineralization peptides was achieved through conjugating a biomineralization peptide (TBP) with an oligomerization peptide (p53Tet) that precisely multimerizes into well-defined frameworks. The oligomeric biomineralization peptide (TBP–p53Tet) showed high binding specificity for silver nanoplates compared to spherical nanoparticles. We used the ratio of the equilibrium constant of TBP–p53 peptides for nanoparticles and nanoplates to quantify the binding specificities for silver nanoplates. The binding specificities of TBP–p53Mono, TBP–p53Di and TBP–p53Tet were 0.90, 1.23 and 1.74, respectively. The high-valent oligomer showed strong specificity for silver nanoplates; indicating that TBP peptides were fixed in suitable positions on the tetramer to interact with silver nanoplates in a coordinated manner. Capping agents show binding affinity for a specific surface of the metal and control the metal nanostructure by regulating the rate of crystal growth^31, 32^.

**Figure 6:**
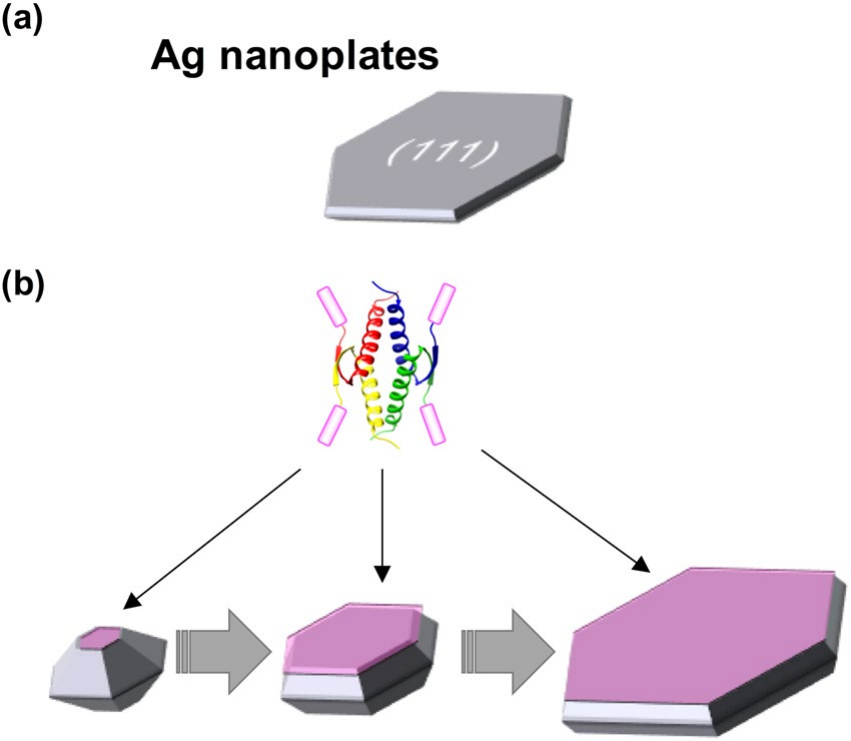
(**a**) Silver nanoplates are mainly enclosed by {111} surfaces. (**b**) A proposed nanostructure control process by using TBP-p53Tet. The TBP-p53Tet recognizes and binds to the silver {111} surfaces, and reduce the rate of crystal growth on the {111} surfaces.

Furthermore, silver specific TBP–p53Tet formed highly symmetric hexagonal nanoplates that were enclosed by {111} facets (Figure S5). It is reported that surfactants specifically bind to {111} surfaces of the truncated triangular silver nanoplates^27, 33^. Therefore, it seems that TBP–p53Tet binds to silver {111} surfaces and reduces the rate of crystal growth on the {111} surfaces (Fig. 6). In addition, TBP–p53Tet controlled the silver nanostructure more precisely than TBP–p53Di. The tertiary structure of TBP–p53Di is almost the same as half of TBP–p53Tet^26^. Therefore, the relative orientation of two TBP peptides conjugated to the oligomer is also the same. These results suggest that TBP–p53Tet binds to silver {111} surfaces via more than two TBP peptides conjugated to an oligomer. Furthermore, the silver {111} surfaces are flat, indicating that a minimum of three TBP peptides were required to regulate the silver nanostructure using TBP–p53Tet.

Interestingly, the TBP–p53Tet regulated the silver nanostructure formation without being consumed. This suggests that TBP–p53Tet acts as a nanoplate-growth catalyst to regulate silver nanostructure formation. The fact that the TBP–p53Tet was not consumed during the formation of the silver nanoplates, indicates that the peptide is not incorporated in the silver nanoparticles, which is different from the formation processes of biomineral structures like bones^34^ and what was previously observed when a palladium binding biomineralization peptide was fused to the p53Tet peptide^22^. In the case of the palladium specific peptide, the tetrameric fusion peptide was incorporated into the nanostructure and formed coral like structures. These results also suggest that TBP–p53Tet regulates the silver nanostructure by binding to specific silver crystal surfaces, restricting free crystal growth, which leads to the formation of hexagonal silver nanoplates.

Analysis of the reaction parameters suggests that there are several factors that determine the type, shape and size of silver nanostructures. The highest proportion of nanoplates was observed at 2 μM TBP–p53Tet, and the nanostructure size decreases with increasing peptide concentration. These results indicate that TBP–p53Tet initially binds to the {111} surfaces, as described above, but excess TBP–p53Tet will bind to other crystal faces after the {111} surfaces are fully occupied. It is known that capping agents bind in an equilibrium manner to inorganic surfaces and are not incorporated into the nanocrystals. In addition, some capping agents help to form ordered metal nanostructures by hindering the crystal growth on the metal surfaces^35, 36^. The TBP–p53Tet peptide is likely to control silver nanostructure formation in a similar manner. The suitable binding affinity and specificity of biomineralization peptides to the metal surface are key factors in regulating inorganic nanostructure formation. These factors can be fine-tuned by using the method of oligomeric biomineralization peptide (e.g. selection of peptide binding target, valence and orientation).

In conclusion, our results demonstrate that the silver biomineralization peptides have enhanced surface-specificity, and regulate the crystal growth process through capping of specific crystal surfaces when coupled to the p53 tetramerization domain. Consequently, the oligomerization state of the biomineralization peptide can also be a major determinant of nanostructure morphology. Although additional experiments are required to precisely elucidate this structural control mechanism, these studies provide new insights into nanostructure control using oligomeric biomineralization peptides, including how they can function as catalysts to promote silver nanostructure formation.

## Methods

### Expression and purification of peptide

The sequence encoding for the TBP–p53Tet fusion peptide (TBP residues RKLPDAGG followed by human p53 residues 324–358) were ordered as oligonucleotides (Integrated DNA Technologies) with flanking BamHI/EcoRI restriction enzymes sites, which were then 5’ phosphorylated, annealed and cloned into a modified pET-15b vector where the thrombin protease cut site was replaced with a Tobacco Etch Virus (TEV) protease cut site. Constructs were verified by DNA sequencing.

The His-tagged TBP–p53Tet peptide was expressed in *Escherichia coli* host strain BL21(DE3). The cells were grown at 37 °C for 20 h. The cells were harvested by centrifugation and resuspended in binding buffer (20 mM phosphate (pH 7.4), 0.5 M NaCl, 50 mM imidazole, 4 M urea). The cells were then lysed by being passed through a French press and centrifuged at 12,000 × g for 60 min. The supernatant from the centrifugation was incubated for 1 h with TALON (Co^2+^) resin at room temperature. After incubation, the resin was collected by centrifugation and washed with binding buffer. The peptide was eluted off the resin by adding the elution buffer (20 mM phosphate (pH 7.4), 0.5 M NaCl, 0.5 M imidazole, 4 M urea) to the resin, and the fractions were collected. The eluted peptide was incubated with 100 units of TEV protease^37^ to remove His-tag from TBP–p53Tet peptide. The protease treated peptide was further purified by C_18_ reverse-phase chromatography. The eluted peptide was incubated with Cosmosil 140C_18_-OPN resin, and washed by 0.05% TFA/H_2_O. The peptide was eluted off the resin by adding 50% MeCN/H_2_O (0.045% TFA). Peptide concentration was measured spectrophotometrically using the extinction coefficient for the TBP–p53Tet peptide, ε_280_ = 1490 M^−1^ cm^−1^, which corresponds to a single tyrosine.

### Peptide synthesis and purification

The TBP peptides conjugated with p53 tetramerization domain mutants composed of residues 324–358 of the p53 protein were synthesized as described previously^38^. Briefly, these peptides were synthesized using an Applied Biosystems 433 A automated peptide synthesizer utilizing the standard Fmoc synthetic strategy on a rink amide resin. The cleaved peptide obtained after treatment with reagent K (9 mL trifluoroacetic acid (TFA), 0.5 mL milli-Q, 0.5 mL phenol, 0.5 mL thioanisole and 0.25 mL of ethanedithiol) was purified using a Shimadzu LC-6AD HPLC equipped with 22 × 250 mm Vydac C8 column with a binary gradient of buffered MeCN/H_2_O as the solvent system. The purified peptides were characterized using an Applied Biosystems Voyager 4379 MALDI-TOF MS. Peptide concentrations were measured spectrophotometrically similar to the procedure described above.

### Binding affinity analysis by quartz crystal microbalance (QCM)

Gold-coated AT cut QCM electrodes with a fundamental resonant frequency of 27 MHz were used. The electrodes were cleaned in piranha solution (98% concentrated H_2_SO_4_/30% H_2_O_2_, 3:1 v/v) for 30 min, followed by rinsing with milli-Q water. The electrode was treated with BSA solution (2 mg/mL, 10 μL) and incubated for 10 min to block the electrode from unspecific binding of peptides. After the blocking treatment, the silver nanostructure solution was dropped on to the electrodes and incubated for 15 min to fix the silver nanostructures by physical adsorption. The silver nanostructures immobilized electrodes were washed with 1 M sodium acetate to remove excess silver nanostructures and citrate. The prepared electrodes were set to AFFINIX Q (initium), and were soaked in buffer (20 mM HEPES-NaOH (pH 7.4), 200 μM L-ascorbic acid, 8 mL) at 20 °C. The peptide solution (1 mM, 5 μL) was added to the buffer after the frequency of electrodes reached equilibrium. We used a Langmuir isotherm to deduce the kinetics of the adsorption by TBP–p53 peptides, and the binding affinity was analysed as previously described^39^.

Silver Nanoplates used for QCM analysis were obtained according to the method previously reported by Zhang *et al*.^40^. Briefly, a 173.6 mL aqueous solution composed of silver nitrate (0.1 M, 180 μL), trisodium citrate (75 mM, 3.6 ml), and H_2_O_2_ (30 wt%, 864 μL) was prepared. Sodium borohydride (NaBH_4_, 100 mM, 1.8 mL) was rapidly injected into this mixture, and the mixture was vigorously stirred at room temperature for 10 min. Spherical Ag nanoparticles were synthesized by the following procedure. 178.9 mL aqueous solution composed of silver nitrate (0.1 M, 180 μL) was adjusted. Sodium borohydride (NaBH_4_, 100 mM, 0.9 mL) was rapidly injected into this mixture, and the mixture was vigorously stirred at room temperature for 10 min. Trisodium citrate (750 mM, 360 μL) was added to the nanoparticles to prevent particles aggregation. The prepared Ag nanostructures were concentrated by centrifugation to use experiments.

### Biomineralization reaction

Peptide solutions with various concentrations were added to 20 mM HEPES-NaOH buffer (pH 7.4), and then silver nitrate solution was added to the buffer (final concentration was 100 μM). The biomineralization reaction was started when a two-fold excess of L-ascorbic acid to silver ions was added to the reaction solution. The solution was incubated for 2 days at several temperatures.

### Electron microscopy

Structure characterization was performed using a scanning transmission electron microscope (Hitachi HD-2000) operating at an acceleration voltage of 200 kV. The observed samples were purified by centrifugation, and the resuspended nanoparticles were placed on a carbon-coated copper grid.

### Quantitative determination of silver nanostructures formed by biomineralization

The amount of silver nanostructures formed by biomineralization reaction was determined by subtracting the amount of unmineralized silver from the initial amount of silver. The amount of unmineralized silver was determined as described previously^41^.

### Determination of peptide consumption

The amount of peptide consumed during the biomineralization reaction was determined by measuring the change of peptide concentration in the reaction solution supernatant before and after the reaction. The levels were determined using a JASCO GULLIVER HPLC equipped with 4 × 250 mm Kanto Chemical C18 column with a binary gradient of buffered MeCN/H_2_O as the solvent system.

## Electronic supplementary material

Supplementary Information
