# Supplementary material for: Oligomerization enhances the binding affinity of a silver biomineralization peptide and catalyzes nanostructure formation
Source: Sci Rep. 2017 May 3;7:1400. doi: 10.1038/s41598-017-01442-8 (PMC5431226; doi:10.1038/s41598-017-01442-8)
Supplement: Supplementary file 1 — Supplementary Information [file 41598_2017_1442_MOESM1_ESM.pdf]

Supplementary Information

Oligomerization enhances the binding affinity of a silver biomineralization peptide and catalyzes nanostructure formation.

Tatsuya Sakaguchi[1], Jose Isagani B. Janairo[1], Mathieu Lussier-Price[2], Junya Wada[1], James G. Omichinski[2], and Kazuyasu Sakaguchi[1*]

[1]Laboratory of Biological Chemistry, Department of Chemistry, Faculty of Science, Hokkaido University, Sapporo 060-0810, Japan, and [2]Département de Biochimie et Médicine Moléculaire, Université de Montréal, C.P. 6128 Succursale Centre-Ville, Montréal, QC H3C 3J7, Canada.

***Correspondence:**

Kazuyasu Sakaguchi
Laboratory of Biological Chemistry, Department of Chemistry,
Faculty of Science, Hokkaido University,
North 10, West 8, Kita-ku, Sapporo 060-0810, Japan.
Email: kazuyasu@sci.hokudai.ac.jp

EXPERIMENTAL SECTION

Gel Filtration Chromatography

The TBP–p53 peptides were separated using a Superdex 75 PC 3.2/30 (GE Healthcare) with a Precision Column Holder (GE Healthcare) in 50 mM phosphate buffer, pH 7.4, 300 mM NaCl. The peptide loading concentrations were 100 μM and the flow rate was 0.1 ml/min at 20 °C, and the effluent was monitored at 280 nm.

Thermal stability analysis by circular dichroism (CD) spectroscopy

For the CD measurements, a Jasco-805 spectropolarimeter was employed using a 1 mm path-length quartz cell. CD spectra were recorded in phosphate buffer (20 mM sodium phosphate (pH 7.4)). For the thermal denaturation studies, spectra were recorded at discrete temperatures from 4 to 96 °C with a scan rate of 1 °C/min. The ellipticity was measured at 222 nm for peptide solution (10 μM), and the unfolding process of the oligomeric TBP–p53 peptides was fitted to a two-state transition model wherein the native tetramer (or dimer) directly converts to an unfolded monomer, as previously described [1,2]. The thermodynamic parameters of the peptides were determined by calculation with the functions described by Mateu *et al* [1]. The T_m and the enthalpy change of unfolding at T_m (ΔH_u^{Tm}) was determined by fitting the fraction of monomer.

Results

Oligomerization state and thermal stability of TBP–p53 peptides

To confirm that the oligomerization is not inhibited by the addition of TBP to oligomeric peptides, the gel filtration chromatography and the circular dichroism (CD) spectra of TBP–p53 peptides were analysed. The elution peaks of TBP–p53Tet and TBP–p53Di were shifted compared to TBP–p53Mono, which indicate oligomer formation (**Figure S1**). In addition, the CD spectra of TBP–p53Tet and TBP–p53Di showed two minima at 208 and 222 nm, which are characteristics of p53 tetramerization domain-like peptide [3] (**Figure S2**). These results indicated that TBP–p53Tet and TBP–p53Di formed p53 tetramerization domain-like tetramers and dimers. TBP–p53Mono showed a negative minimum near 200 nm, which is characteristic of a random coil.

The thermal stability of TBP–p53Tet and TBP–p53Di was investigated by calculating the thermal denaturation curves for each TBP–p53 peptide from changes in the CD ellipticity at 222 nm using a two-state transition mode. The denaturation curves of TBP–p53Tet and TBP–p53Di are shown in **Figure S3**, and the thermodynamic parameters are summarized in **Table S1**. The melting temperatures (T_m) of TBP–p53Tet and TBP–p53Di were 67.3 and 43.1 °C, respectively, and the free energies of unfolding at 20 °C ($\Delta G_u^{20°C}$) of these peptides were 30.5 and 10.7 kcal/mol, respectively. The parameters of TBP–p53Tet were the almost same as those of p53Tet, indicating that attaching the TBP peptide to p53Tet had no effect on the stability of the p53 tetramer. The oligomer stability of TBP–p53Di was lower than that of TBP–p53Tet, but almost all of TBP–p53Di formed an oligomer at 20 °C (**Table S2**).

Silver nanostructures formed in the absence of p53 peptides or in the presence of only the TBP peptide.

In the absence of p53 peptides or in the presence of only the TBP peptide, disordered

spherical silver nanostructures were formed (**Figure S4**). These nanostructures are completely different from those formed with the oligomeric TBP–p53 peptides. These data suggest that the oligomerization of biomineralization peptides is required to control the morphology of silver nanostructures.

Crystal plane of silver nanoplate formed with TBP–p53Tet

The silver nanoplate surface formed by TBP–p53Tet was observed by using high-resolution TEM (**Figure S5**). The atomic arrangement of 6-fold symmetry and the lattice fringe spacing of 2.36 Å can be indexed to the {111} reflection of face-centered cubic (fcc) Ag [4].

References

1 Mateu, M. G. & Fersht, A. R. Nine hydrophobic side chains are key determinants of the thermodynamic stability and oligomerization status of tumour suppressor p53 tetramerization domain. *EMBO J.* **17**, 2748-2758 (1998).

2 Johnson, C. R., Morin, P. E., Arrowsmith, C. H. & Freire, E. Thermodynamic analysis of the structural stability of the tetrameric oligomerization domain of p53 tumor-suppressor. *Biochemistry* **34**, 5309-5316 (1995).

3 Kamada, R., Nomura, T., Anderson, C. W. & Sakaguchi, K. Cancer-associated p53 tetramerization domain mutants qiantitative analysis reveals a low threshold for tumor suppressor inactivation. *J. Biol. Chem.* **286**, 252-258, (2011).

4 Downs, R. T. & Hall-Wallace, M. The American mineralogist crystal structure database. *Am. Mineral.* **88**, 247-250 (2003).

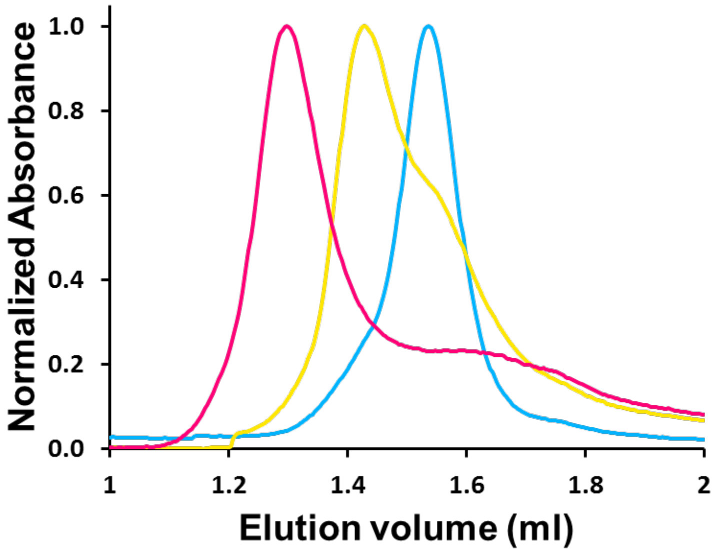

Figure S1. Gel filtration chromatogram of TBP–p53Tet (magenta), TBP–p53Di (yellow) and TBP–p53Mono (blue). The oligomeric states of TBP–p53 peptides were analysed by gel filtration on a Superdex 75 PC3.2/30 using a sodium phosphate buffer (pH 7.4) at 20 °C.

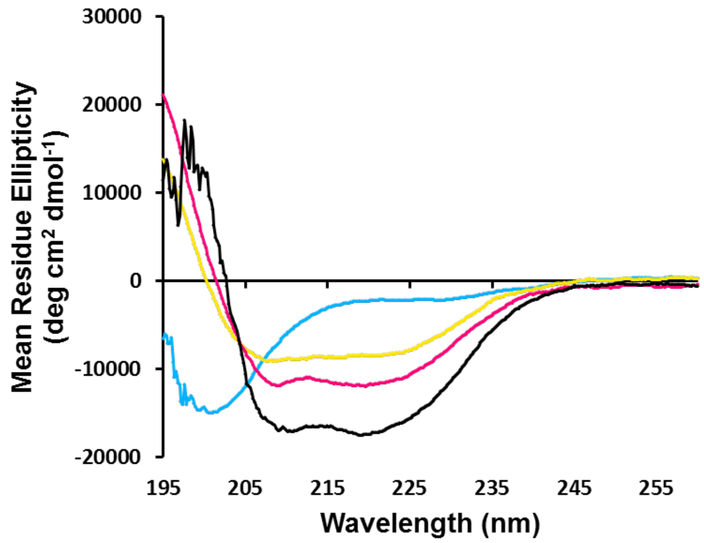

Figure S2. CD spectra of TBP–p53 peptides and p53 tetramerization domain peptide. CD spectrum of TBP–p53Tet (magenta) and TBP–p53Di (yellow) was quite similar to p53Tet (black). TBP–p53Mono (blue) showed the typical spectrum of random coil. The peptides were in 20 mM phosphate buffer, pH 7.4, at 20 °C. Peptide concentration was 10 µM.

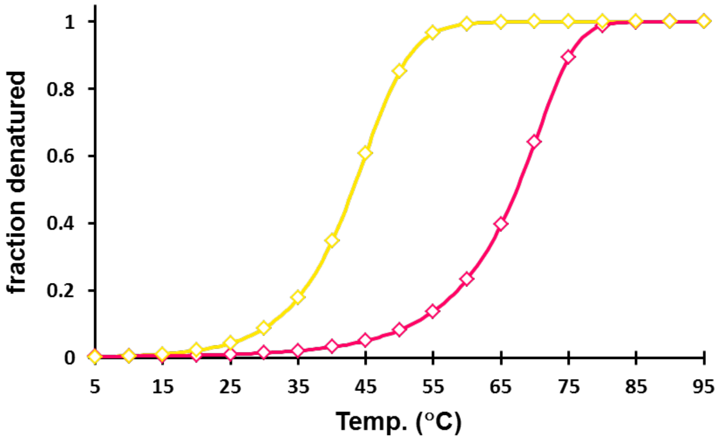

Figure S3. Thermal denaturation of TBP–p53Tet (magenta) and TBP–p53Di (yellow). Thermal denaturation of the peptides was analysed by measuring the ellipticity at 222 nm for 10 μM peptide solutions in 20 mM phosphate buffer, pH 7.4 over the range of 4 °C to 96 °C, with a scan rate of 1 °C per minute.

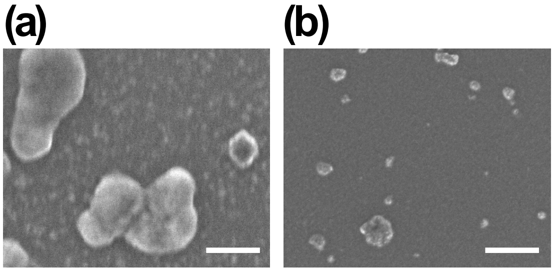

Figure S4. Representative SEM images of silver nanostructures (a) in the absence of peptides and (b) in the presence of the TBP peptide. The scale bar is 100 nm.

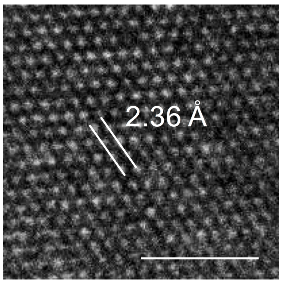

Figure S5. High-resolution TEM images of silver nanostructure surface formed by using TBP–p53Tet. The scale bar is 2 nm.

Table S1. Thermodynamic parameters of the oligomeric peptides

peptide	T_m °C	$\mathit{\Delta} H_u^{Tm}$ kcal/mol	$\mathit{\Delta} G_u^{20°C}$ kcal/mol
TBP–p53Di	43.1	64.4	10.7
TBP–p53Tet	67.3	112.5	30.5
p53Tet	67.4	110.9	30.3

T_m: transition temperature, $\mathit{\Delta} H_u^{Tm}$: variation in the enthalpy of unfolding at T_m, $\mathit{\Delta} G_u^{Tm}$: variation in the Gibbs free energy of unfolding at 20 °C.

Table S2. The percentage of the peptide in denatured state at each temperature.

peptide	temperature (°C)									
	5	10	20	30	40	50	60	70	80	90
TBP–p53Di	0.4	0.6	2.3	8.9	34.9	85.3	99.4	100.0	100.0	100.0
TBP–p53Tet	0.5	0.5	0.8	1.5	3.3	8.3	23.5	64.2	98.9	100.0
p53Tet	0.5	0.6	0.9	1.6	3.4	8.5	23.5	63.4	98.7	100.0
